# Deep Learning Solution for Quantification of Fluorescence Particles on a Membrane

**DOI:** 10.3390/s23041794

**Published:** 2023-02-05

**Authors:** Abdellah Zakaria Sellam, Azeddine Benlamoudi, Clément Antoine Cid, Leopold Dobelle, Amina Slama, Yassin El Hillali, Abdelmalik Taleb-Ahmed

**Affiliations:** 1 Institute of Applied Sciences and Intelligent Systems, National Research Council of Italy, 73100 Lecce, Italy; 2 Laboratoire de Génie Électrique, Faculté des Nouvelles Technologies de l’Information et de la Communication, Université Ouargla, Ouargla 30000, Algeria; 3 Linde Laboratories, California Institute of Technology, Pasadena, CA 91125, USA; 4 Faculty of Humanities and Social Sciences, Mohamed Khider University of Biskra, Biskra 07000, Algeria; 5 Institut d’Electronique de Microélectronique et de Nanotechnologie (IEMN), UMR 8520, Université Polytechnique Hauts de France, Université de Lille, CNRS, 59313 Valenciennes, France

**Keywords:** mgLAMP, YOLOv5, GFC, SARS-CoV-2, TTA, CSPnet

## Abstract

The detection and quantification of severe acute respiratory syndrome coronavirus-2 (SARS-CoV-2) virus particles in ambient waters using a membrane-based in-gel loop-mediated isothermal amplification (mgLAMP) method can play an important role in large-scale environmental surveillance for early warning of potential outbreaks. However, counting particles or cells in fluorescence microscopy is an expensive, time-consuming, and tedious task that only highly trained technicians and researchers can perform. Although such objects are generally easy to identify, manually annotating cells is occasionally prone to fatigue errors and arbitrariness due to the operator’s interpretation of borderline cases. In this research, we proposed a method to detect and quantify multiscale and shape variant SARS-CoV-2 fluorescent cells generated using a portable (**mgLAMP**) system and captured using a smartphone camera. The proposed method is based on the YOLOv5 algorithm, which uses CSPnet as its backbone. CSPnet is a recently proposed convolutional neural network (CNN) that duplicates gradient information within the network using a combination of Dense nets and ResNet blocks, and bottleneck convolution layers to reduce computation while at the same time maintaining high accuracy. In addition, we apply the test time augmentation (TTA) algorithm in conjunction with YOLO’s one-stage multihead detection heads to detect all cells of varying sizes and shapes. We evaluated the model using a private dataset provided by the Linde + Robinson Laboratory, California Institute of Technology, United States. The model achieved a mAP@0.5 score of 90.3 in the YOLOv5-s6.

## 1. Introduction

As the COVID-19 pandemic caused by the SARS-CoV-2 virus becomes endemic and with the frequent apparition of new variants of the virus, monitoring SARS-CoV-2 occurrence is still a key tool to detect new variants of concerns, find infection hotspots, and prevent wide shutdowns. Li et al. [1] Wastewater Based Epidemiology (WBE) combined with Molecular Diagnostics (MD) such as DNA or RNA detection using polymerase chain reaction (PCR) or loop-mediated isothermal amplification (LAMP) have shown to be powerful tools to assess the prevalence of COVID-19 (and other diseases) in a specific population without the need for individual testing (Bivins et al. [2]; Peccia et al. [3]; Polo et al. [4], Sims & Kasprzyk-Hordern [5,6]). In principle, it is possible to equip clinical MD laboratories to perform environmental water analyses, but such laboratories are often available only in urban (high population density) areas of high-income countries and rural areas and remote communities suffer from limited access to molecular testing [7]. Therefore, MD tools for WBE are often underutilized in rural settings, and outbreaks are not detected early enough. To minimize the necessity of specialized equipment, highly skilled personnel, and labor-intensive laboratory procedures, automated and inexpensive point-of-sampling testing methods must be developed for large-scale environmental surveillance. DNA or RNA amplification from MD technologies is detected via signal amplification of a direct physical response such as an increase of luminescence intensity [8]. The detection relies on quantification of the signal intensity (i.e., analog response) directly correlated to the original concentration of the targeted DNA or RNA. Examples of such commercial systems used in microbial or viral analysis of wastewater include the LuminUltra [9] or Cepheid system [10].

Counting reacted and unreacted microscopic cells and particles counting is an essential step employed in many MD techniques requiring discretization of reaction chambers. For instance, the MD-based approach utilized by Zhu et al. [11] named mgLAMP quantifies the amount of RNA-amplicon dots, corresponding to whole viral particles being retained by a small pore size (80 nm) membrane. This approach relies on the loop-mediated isothermal amplification (LAMP) reaction in a gel matrix with the addition of fluorescence probes and quenchers that will illuminate at 517 nm under 492 nm excitation light.

**Figure 1:**
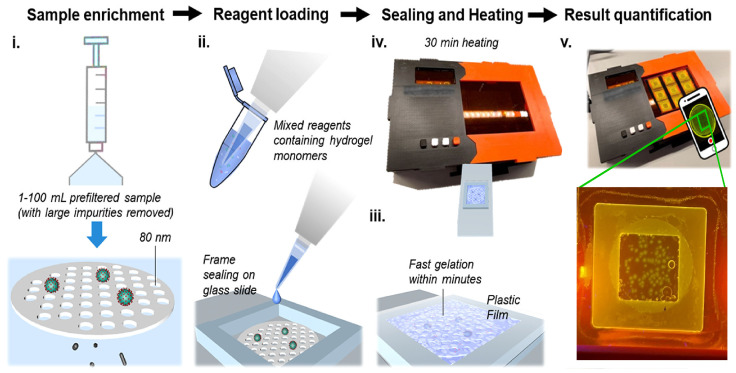
Data retriving process from the mgLAMP (**i**) Filtration. (**ii**) Reagent loading. (**iii**) Sealing. (**iv**) Incubation. (**v**) Imaging.

The mgLAMP system is a one-step method for detecting SARS-CoV-2 by using a microfiltration membrane, PEG hydrogel, and fluorescent probes. The system is designed to specifically capture and amplify SARS-CoV-2 RNA, the genetic material of the virus that causes COVID-19 from a sample, which results in high sensitivity and specificity. The mgLAMP workflow in Figure 1i begins by filtering the sample through a PCTE membrane to retain the virus particles (SARS-CoV-2) and eliminate other molecules that may inhibit the RT-LAMP reaction. Then, in Figure 1ii, a mixture of PEG hydrogel monomers and RT-LAMP reagents is added on top of the virus-loaded membrane on a glass slide with a Frame-Seal incubation chamber in Figure 1iii. The hydrogel crosslinks and undergoes an RT-LAMP reaction in the mgLAMP prototype device in Figure 1iv. The reagents include Triton X-100 for in-assay viral lysis and a target-specific QUASR probe which facilitates fluorescence amplicon dot formation in the hydrogel. The fluorescent signals are captured using a smartphone for analysis in Figure 1v.

In recent years, computer vision-based methods have been proposed for the detection and identification of cells in microscopy [12]. These methods include traditional techniques such as intensity thresholding, morphological filtering, and deformable model fitting [13,14,15]. However, traditional counting methods can be challenging due to the varying size and shape of cells. To overcome this limitation, researchers have adopted AI-based methods such as machine learning-based classifiers [16,17,18], deep convolutional networks [16], and object detection models such as RCNN [19], Fast-RCNN [20], FPN [21], SSD [22], YOLO [23]. These methods have proved to be more efficient and accurate than manual counting, and they also increase the overall throughput of the laboratory by freeing up time for laboratory technicians to process other samples. The YOLOv5 [24,25] model, in particular, is fully written in the PyTorch framework [26], making it more accessible and easier to use in a wide range of development environments. In this article, we present an adaptation of the YOLOv5 model to recognize and quantify multiscale SARS-CoV-2 cells in environmental wastewater using fluorescent reactions from bioluminescent protein in SARS-CoV-2 cells captured using a smartphone camera and generated using a portable Membrane-Based In-Gel Loop-Mediated Isothermal Amplification (mgLAMP) system [11]. The main contributions of our work are summarized as follows:

1. We created a new dataset of high-resolution SARS-CoV-2 RNA images captured using advanced microscopy for training and validation, and test images captured from a mgLAMP device using a smartphone camera.
2. We applied YOLOv5 algorithm combined with Test Time Augmentation (**TTA**) to detect and quantify SARS-CoV-2 fluorescent cells.
3. We developed preprocessing block to extract region of interest based on Hough transform and Cramer rule.

## 2. Proposed Approach

**Figure 2:**
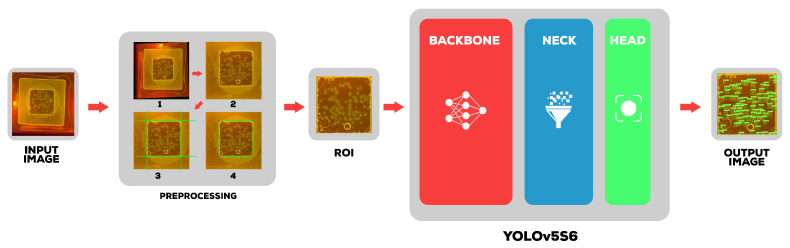
General structure of the proposed approach.

In this section, we provide a detailed explanation of the proposed work starting with the preprocessing and proposed YOLOv5-s6 [27] detection model. As illustrated in Figure 2, the proposed approach is split into two stage.

The first stage is preprocessing, which uses Hough transform, and Cramer rule techniques to align and extract the region of interest from the input image, then feeds it to the second stage, which is YOLOv5-s6 detection, which consists of three parts: the backbone, the neck, and the head.

**Figure 3:**
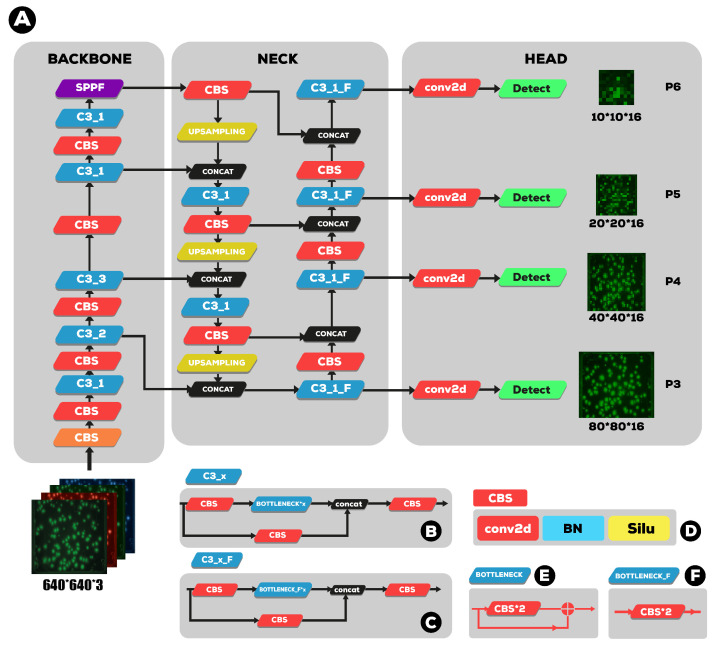
YOLOv5 version 6.1 (**A**): the overall architecture that consists of three main parts: the backbone, the neck, and the head modules. (**B**,**C**) two distinct types of CSP blocks (C3). In (**D**), a key block called CBS is defined, which consists of a Conv layer, a BN layer, and a SILU This CBS block is used in many other blocks (**E**) and two different botllneck blocks (**F**).

The proposed method for detecting and quantifying SARS-CoV-2 fluorescent cells that have been specifically marked with a fluorescent dye or protein in order to detect their presence in environmental water is based on the YOLOv5-s6 a variant of the YOLOv5 model. YOLOv5-s6 includes an additional output layer on 64 strides to detect more large-scale cells in addition to the three existing scales, as schematized in Figure 3. The model is flexible and compact with high real-time detection accuracy.

### 2.1. Preprocessing

**Figure 4:**
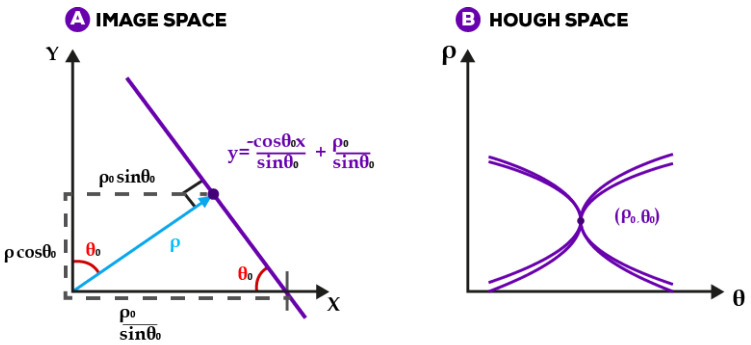
Hough transform converts the Cartesian representation of points in image (**A**) to polar coordinates, as shown in (**B**). By identifying clusters of points with similar polar coordinates, this transformation allows for the detection of lines in the image according to specified parameters.

Capturing high-quality endpoint fluorescence images from the portable mgLAMP incubation chamber using a smartphone camera can be crucial to the final detection and quantification results. However, in the same way, extracting the centre square from a plastic frame mounted in an incubation chamber, as Figure 1v shows, can be time-consuming when done manually, so we proposed a method to automate the extraction of regions of interest using the Hough Transform [28] and Cramer rule. First, we will start by autoaligning the image using Hough transform, a feature extraction technique that converts an image from Cartesian to polar space, as shown in Figure 4. The following Equation (1) represents Hough space.

(1) ρ=xcosθ+ysinθ

We are using the Hough transform in this case to detect lines. A line equation is represented in the Equation (2). The line is represented by the length of that segment ρ and the angle θ that it makes with the *x*-axis in image space, as shown in Figure 4.

(2) $$y=\frac{-x\cos\theta }{\sin\theta }+\frac{\rho }{\sin\theta }$$

The coordinates in image space will be transformed to the point of the form (ρ,θ) in Hough space following the next steps.

**Figure 5:**
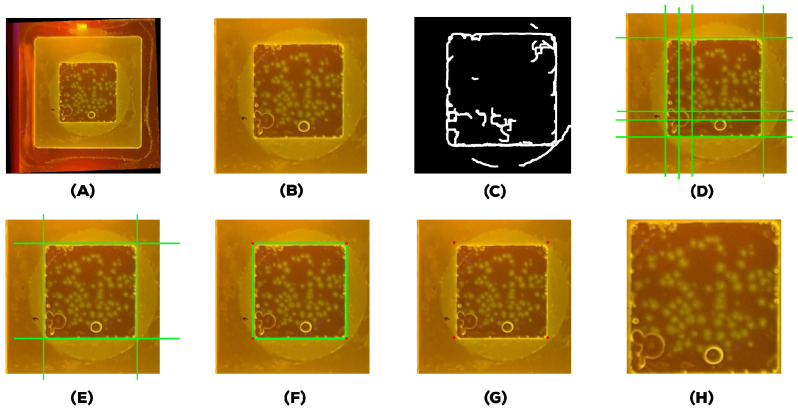
Steps of reprocessing: (**A**) rotated image, (**B**) zoomed image, (**C**) image after applying Canny edge detection, (**D**,**E**) intersection points identified using the Hough transform, (**F**,**G**) point selected for cropping the image, (**H**) cropped image.

1. Applying Canny filter to minimize computational cost and noise and binaries the image, as you can see in Figure 5C, to detect the most relevant lines.
2. Applying probabilistic Hough lines to find lines between 0.1 and 180-degree angles.
3. Rounding the angles from line peaks to two decimal places, we use the angle with the highest occurrence.
4. Rotating the image with the resulting angle as depicted in Figure 5A.

After we apply those steps, we obtain the aligned image (A) in Figure 5. Next, we apply a zoom-in operation on the image. As presented in Figure 5B to eliminate all the possible horizontal and vertical lines that do not belong to the region of interest; then, we apply the Hough transform again to get the nearly horizontal and vertical lines. As shown in Figure 5D, then, we extract the four lines: the top edge line, the left edge line, the right edge line, and the bottom edge line, as you can see in Figure 5E,F.

Following that, we transform the lines described by the Equation (1) into system equations of the following form:

(3) ax+by+c=0

By replacing the coefficients a, b, and c from the general system Equation (3), the equation will become:

(4) $$\begin{array}{c} a=\cos\theta \;b=\sin\theta \;c=-\rho \\ x\cos\theta +y\sin\theta +(-\rho )=0 \end{array}$$

Then, we can apply the Cramer rule to find the system solution, which is the point of intersection (*x*, *y*) between every two lines using the equation below.

(5) $$\left(x,y\right)=\left[\frac{b_{1}c_{2}-b_{2}c_{1}}{a_{1}b_{2}-a_{2}b_{1}},\frac{b_{1}c_{2}-b_{2}c_{1}}{a_{1}b_{2}-a_{2}b_{1}}\right]$$

Finally, after finding the four intersection points (top-left, top-right, bottom-right, and bottom-left), as Figure 5G shows, we take the four points and crop the region of interest from the original image. The resulted image is shown in Figure 5H.

### 2.2. Overview of the YOLOv5 Architecture

**Figure 6:**
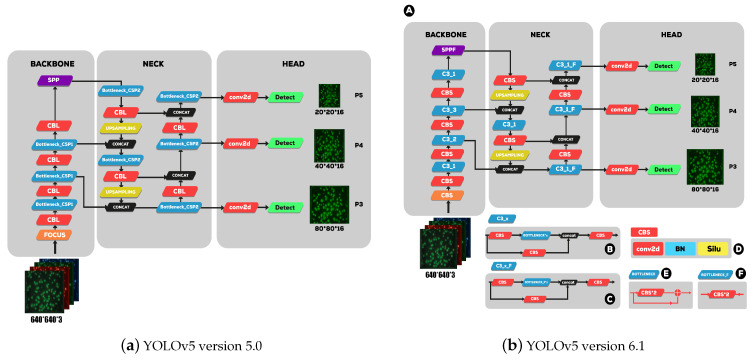
(**a**) YOLOv5 version 5.0 architecture, which consists of three main parts: the backbone, the neck, and the head. (**b**) YOLOv5 version 6.1 (A): the overall architecture that consists of three main parts: the backbone, the neck, and the head modules. (B,C) Two distinct types of CSP blocks (C3). In (D), a key block called CBS is defined, which consists of a Conv layer, a BN layer, and a SILU. This CBS block is used in many other blocks (E) and two different bottleneck blocks (F).

YOLOv5 provides four different scales for their model, S, M, L and X, which stand for Small, Medium, Large, and Xlarge, respectively. Each of these scales applies a different multiplier to the depth and width of the model, meaning that the overall structure of the model remains constant, but the size and complexity of each model are scaled. The basic structure of the previous YOLOv5 is largely divided into the backbone, the neck, and the detection head, as Figure 6a,b shows. The backbone consists of a convolutional neural network aggregating image features at various particle sizes. In contrast, the neck consists of a series of layers that mix and combine image features to deliver prior to prediction, and the head consumes features from the neck (PAnet) and takes box and class prediction steps. There are two major differences between previous YOLOv5 version 5.0 (as illustrated in Figure 6a and current (YOLOv5 version 6.1 as shown in Figure 6b).

**Figure 7:**
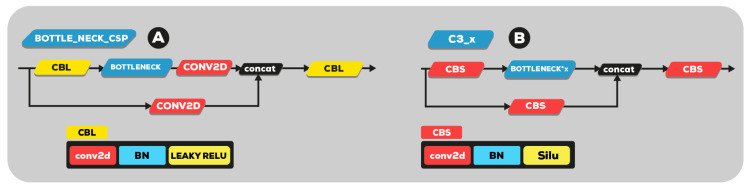
(**A**) YOLOv5 version 5.0 bottleneck CSP and (**B**) new C3 module.

The same author transforms the bottleneck CSP (bottleneck layer) module, as illustrated in Figure 7A, into the C3 module Figure 7B. Its structure and function are the same as CSP’s and include three standard convolution layers and multiple bottleneck modules. The differences between the C3 and CSP modules is that the Conv module after residual output is removed, and the activation function in the standard convolution module after the Concat module is changed from LeakyRelu to SiLU.

**Figure 8:**
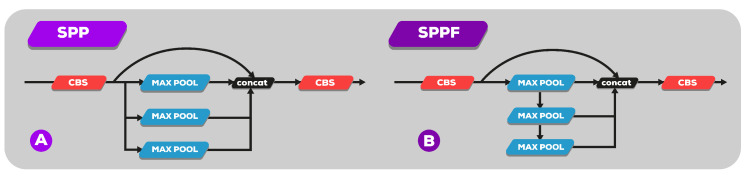
(**A**) SPP module for YOLO series. (**B**) SPPF module.

The SPP layer in Figure 8A was replaced by the SPPF layer. SPPF [27], instead of SPP [29], can reduce flops, run faster, and realize local features. The SPPF network structure is shown in Figure 8B. The fusion of local and full moment features is conducive to the large difference in target size in the image to be detected in the security wear recognition image. It can enrich the expression ability of the feature map. Therefore, accuracy can be greatly improved, especially for complex multitarget detection.

**Figure 9:**
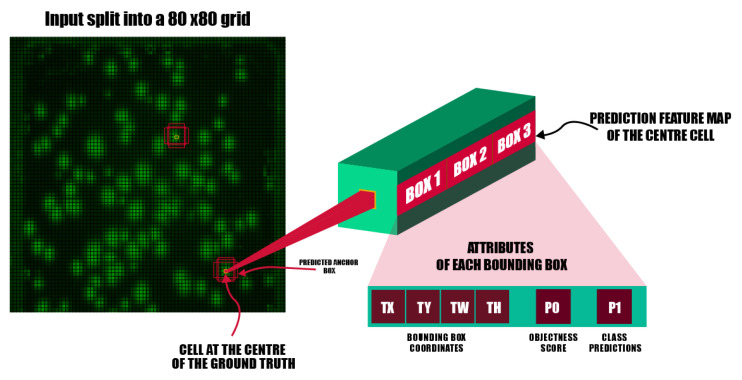
Example of a YOLOv5 workflow when applying a 80 × 80 grid to the input image. The input image is split into 3600 grid cells. Each grid cell predicts 3 bounding boxes and their objectness score along with their class predictions.

YOLOv5 uses the YOLOv3 [30] anchor-based head. The detection network is mainly used for the final detection part of the model. It applies anchor boxes on the feature map output from the previous layer and outputs a vector with the category probability of the target object, the object score, and the position of the bounding box surrounding the object. Those are:

- Coordinates of bounding boxes—Similar to previous detectors, YOLO predicts four coordinates for each bounding box (Tx, Ty, Tw, Th) as shown in Figure 9, where *x* and *y* are set to be offsets of a cell location.
- Objectness score (P0)—indicates the probability that the cell contains an object. The abjectness score is passed through a sigmoid function to be treated as a probability with a value range between 0 and 1. The objectness score is calculated as follows:
  (6) AP=Pr(containinganobject)×IoU(pred,truth)
- Class prediction: If the bounding box contains an object, the network predicts the probability of K number of classes. Where K is the total number of classes in our problem, we have one class k = 1.
- The detection network of YOLOv5-s architecture is composed of three detection layers. Their input is a feature map with dimensions of 80×80, 40×40, and 20×20, respectively. Each detection layer used to detect the image objects of different sizes outputted an 18-channel vector first ((1 class + 1 class probability + 4 surrounding box position coordinates) × 3 anchor boxes). Then, the predicted bounding boxes and categories of the targets in the original image were generated and labeled, therefore implementing the detection of the GFC targets in the image.

### 2.3. Adaptive Anchor Box

The creator of YOLOv5 highlighted a notable change in engineering. In YOLOv2, Joseph Redmon [31] proposed the anchor box structure and a technique for picking anchor boxes of size and form that closely approximated the training set’s ground truth bounding boxes. Applying the k-mean clustering method with varying parameters (containing 80 classes) and using them as the default reduced training time and increased the accuracy of the network. However, when these five anchor boxes were applied to a unique dataset (including a class that is not one of the 80 classes in the COCO dataset), they could not swiftly adapt to the ground truth bounding boxes of this unique dataset. For example, if the dataset contains images of small objects such as coins, the bounding boxes used for detection should be relatively small and compact, allowing the algorithm to accurately locate and identify the coins within the image. In contrast, a long bounding box would be less suitable for small objects as it would be too big and may not be able to accurately detect the objects. To overcome this issue, computer vision experts often ran the k-mean clustering method on the unique dataset first to obtain the best-fit anchor boxes for the data. Glenn Jocher recommended that the anchor box selection method be included in YOLOv5. As a result, the network does not need to evaluate any of the datasets to be used as input; instead, it will automatically "learn" and utilize the optimal anchor boxes for that dataset during training.

### 2.4. Test-Time Augmentation (TTA)

**Figure 10:**
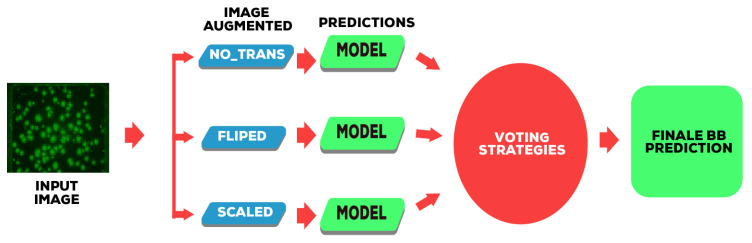
Workflow of TTA ensemble algorithm. Three methods have been applied to detect the objects in the original image.

Data augmentation [32,33] is a technique that was widely used to train deep learning models. It involved creating additional training samples from the original training dataset using transformations that did not change the data’s class. A variation of data augmentation, known as test-time augmentation [34], was used for the test dataset. This approach generated random variations of the test image, made predictions for each one, and then produced an ensemble of those predictions. In image classification, test-time augmentation (TTA) was straightforward, as it involved executing various picture transformations (such as flips, rotations, color transformations, etc.), making predictions for each one using a specific model, and then returning the ensemble of those predictions. However, TTA is not as simple when it came to object detection because some transformations, such as flips or crops, changed the position of the objects. In more detail, given an image and a list of detections, detection transformation is an operation that returned a transformed image and a list of detections. We applied each image transformation to obtain new images as a result, as shown in Figure 10. Subsequently, we detected the objects in each image using the object detection model and produced lists of detections. For each result, we applied a detection transformation that returned a list of detections in the correct position for the original image. Finally, we ensemble the predictions using one of the three voting strategies (NMS (nonmaximum suppression), affirmative, consensus, or unanimous) [35].

## 3. Performance Evaluation

In this section, we first describe the datasets used in our experiments. Then, we give the evaluation metrics used. We present the performance of our proposed scheme as well as that of other competing methods. Finally, we provide the analysis and discussion of the obtained results.

### 3.1. Dataset

**Figure 11:**
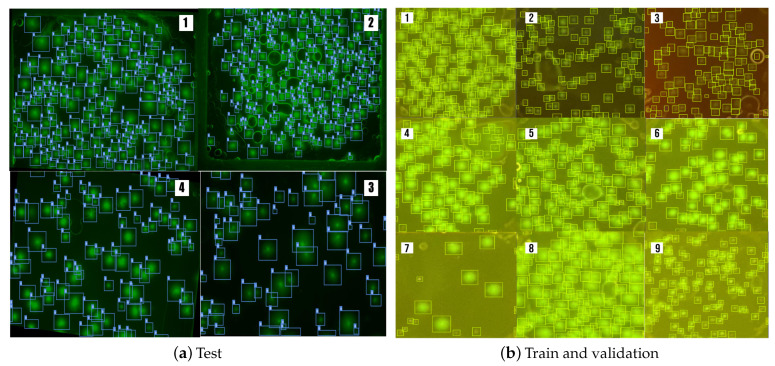
(**a**) Examples of green fluorescent cell images that have been captured from the Leica DMi8 fluorescence microscope and meticulously annotated. (**b**) Examples of green fluorescent cell images that have been captured from mgLAMP using a smart phone camera and meticulously annotated by scientists.

The dataset used in this paper is a private dataset that Caltech Institute of Technology Linde + Robinson Laboratory has provided. The dataset contains images of multiscale SARS-CoV-2 fluorescent cells, as shown in Figure 11a,b. The dataset contains 241 manually annotated pictures captured using a Leica DMi8 fluorescence microscope (Leica Microsystems, Wetzlar, Germany) and smartphone camera images captured using a Google Pixel 4 set in night mode.

The images from the microscope were split into two ensembles: The training ensemble consisted of 131 images with 3930 annotated cells. The validation ensemble consisted of 33 images with 1332 annotated cells. An example of the training dataset can be seen in the Figure 11a.

The test dataset captured from a portable mgLAMP device using a Google Pixel 4 smartphone camera contains 77 images with 4666 fluorescent dots. The Figure 11b illustrates examples of the test dataset.

To evaluate the effectiveness of the GFC (Green Fluorescent Cells) detection model, in this work, we propose precision (P), which is precision rate, recall rate (R), and mean average precision (mAP). P and R can be expressed as follows:

(7) $$P=\frac{TP}{TP+FP}$$

(8) $$R=\frac{TP}{TP+FN}$$

True positives (TP), false positives (FP), and false negatives (FN) are positive samples with proper classification, negative samples with erroneous classification, and so on, and positive samples with incorrect classification. If the IoU between the detection box and the GFC bounding box is greater than 0.5, the detection box is marked as TP. Otherwise, the detection box is marked as FP. If the GFC bounding box does not have a matching detection box, it is marked as FN. TN is not required in this binary classification problem, where the foreground is always determined for GFC detection. TP and FP are separately the number of GFC detected correctly and incorrectly, and FN is the number of undetected GFC. AP refers to the average recall rate of the GFC detection in the range of 0 to 1. Therefore, higher AP (See Equation (9)) means higher accuracy of the network; mAP (See Equation (10)) is the average accuracy of the mean, which implies that the AP value of each category is added together and then divided by all categories, yielding the average value. However, in our case, the map is the same as AP because we have only one class, which is GFC.

(9) $$AP=\int_{0}^{1}P\left(R\right)\;dR$$

(10) $$MAP=\frac{1}{Q_{r}}*\sum_{q=Q_{r}}AP\left(q\right)$$

$Q_{r}$ = Number of classes

### 3.2. Implementation

The experiment was performed on a Google Colab notebook with the following specifications: NVIDIA Tesla P100 graphics processor (16 GB of memory), and 130 GB of memory (RAM: 32 GB), with the Ubuntu operating system. The network in this study was trained using a stochastic gradient descent (SGD) optimizer. The batch size for model training was set to 16, and the BN layer performed regularization to update the model’s weight each time. The momentum factor (momentum) was set to 0.937 and the weight decay rate (decay) to 0.0005. The training epoch count was set at 300, with the first three epochs as warm-up epochs to help our network slowly adapt to the data by making a few updates with a low learning rate at the beginning of training. After this warm-up, we used the regular learning rate (schedule) to train our model to convergence. Table 1 displays a summary of the values of the hyperparameters used in this experiment.

### 3.3. Loss Functions of Bounding Box

**Figure 12:**
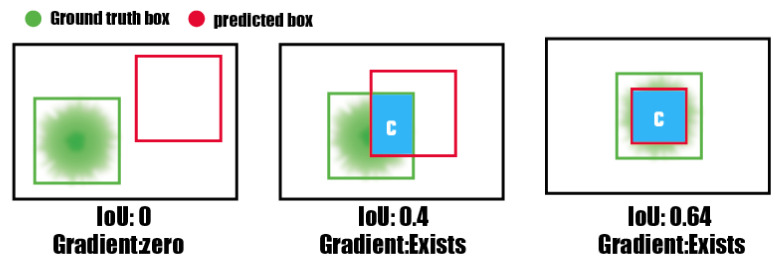
Example of intersection area (IoU). ’C’ represents the Intersection, which is the area of overlap between the ground truth and prediction.

IoU [36] stands for intersection over union, and it is a common indicator in target detection. IoU is produced by the interaction between the predicted box and the ground truth box. That is, it is a value representing the size of the predicted Bounding Box and Ground Truth in the field of object detection a value between 0 and 1 (as shown in Figure 12). At the same time, if the loss is 0, there is no gradient back and learning and training are not possible.

**Figure 13:**
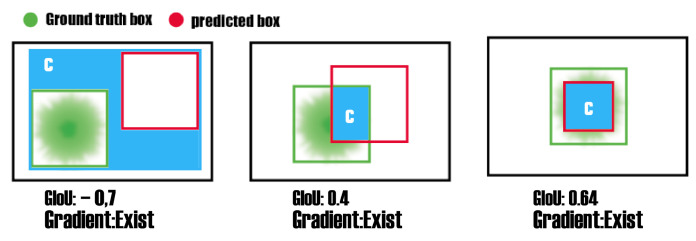
Various GIoU situations with values in each and an indicator of gradient presence in each case based on ’C’, which represents the Box Displacement between GT and Prediction.

The Generalized Intersection over Union (GIoU) is a measure of similarity between two bounding boxes, commonly used in object detection tasks. It extends the traditional Intersection over Union (IoU) metric by taking into account the additional area of union beyond the intersection. The GIoU metric is calculated by first finding the intersection between box A (the ground truth) and box B (the prediction), represented by the blue box. Then, subtracting the ratio of the additional area of union between box A and B (the areas that do not overlap) from the intersection of the two boxes divided by the union of the two boxes. When 1-GIoU is used as a loss function in object detection, it allows for more accurate bounding box predictions by iteratively expanding the B box area to overlap with the GT and then reducing the B box area to increase IoU. This is because the loss function is designed to minimize the difference between the predicted box and the ground truth by adjusting the coordinates of the predicted box. As a result of this, the model’s prediction gets better with each iteration, thus resulting in more accurate bounding box predictions (as shown in Figure 13).

This can improve the gradient vanishing problem for nonoverlapping boxes, but there was a problem that the convergence rate was slow and the box was predicted incorrectly. To solve this problem, we use CIoU (Complete-IoU). CIoU loss bounding box regression uses three geometric factors:

- Overlap area between the predicted box and the ground truth bounding box-IoU loss.
- The central point between the predicted box and the ground truth bounding box-DIoU loss.
- An aspect ratio of the predicted box and the ground truth box.

**Figure 14:**
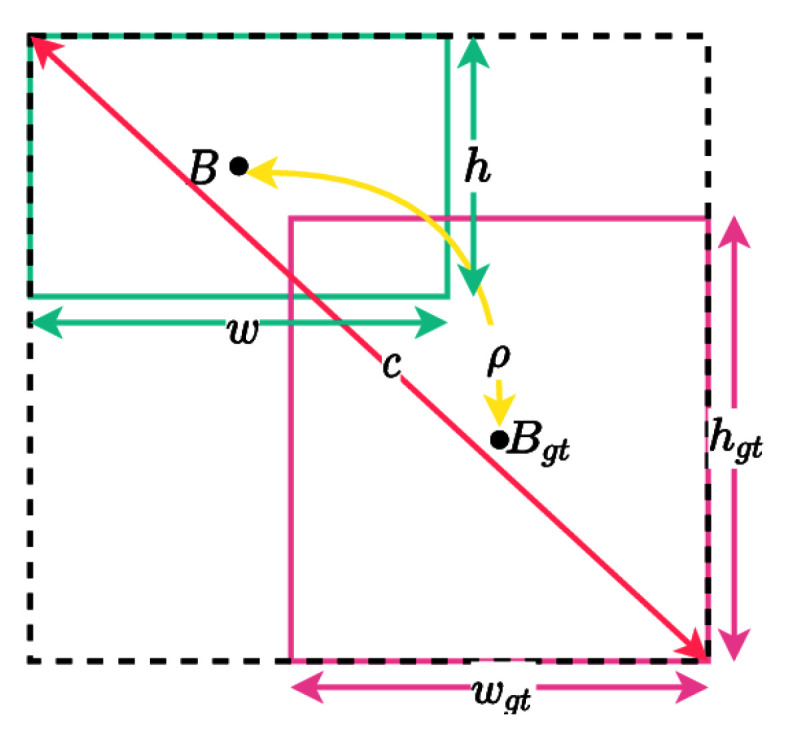
CIoU situations. (ρ): Euclidean distance between two centre points. (*B*): represents the centre points of the prediction box. ($B_{g_{t}}$): represents the centre points of the ground truth box. (α): The weight function. c: represents the diagonal distance of the minimum closure region that can contain both prediction frame and real frame. (υ): measures the similarity of aspect ratio.

As CIoU loss uses complete geometric factors as you can see in Figure 14, it converges faster than GIoU loss [37]. It improves average precision (AP) and average recall (AR) for object detection. Equation (11) represent the CIoU loss.

(11) $$L_{CIoU}=1-IoU+\frac{\rho^{2}\left(B,B_{g_{t}}\right)}{C^{2}}+\alpha \upsilon$$

### 3.4. Results and Discussion

We trained our model for 300 epochs on 131 fluorescence microscopy pictures with a size of 640×640×3. Then, we validated our models (YOLOv5-s and YOLOv5-s6) on 33 validation images from the fluorescence microscopy image dataset to compare them, yielding the findings shown in Table 2.

**Figure 15:**
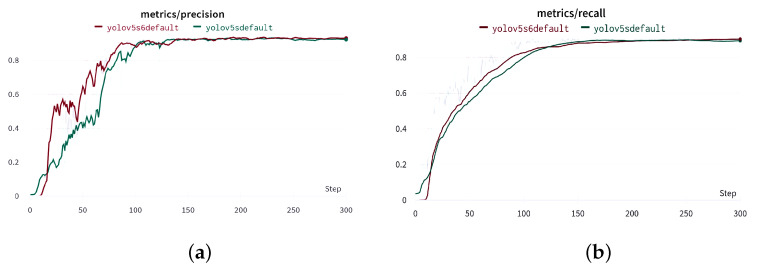

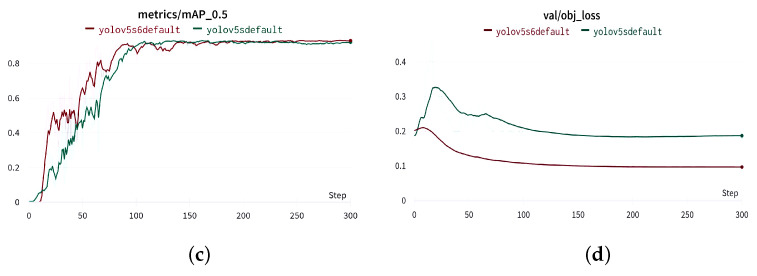
Results of validation phase using YOLOv5-s and YOLOv5-s6. (**a**) precision, (**b**) recall, (**c**) mAP@0.5 and (**d**) objectness loss.

We observe from Figure 15 that YOLOv5-s6 produces better results than YOLOv5-s. It has faster convergence and lower objectness loss, which means that YOLOv5-s6 has a bigger probability that an object exists in a proposed region of interest than YOLOv5-s, and that is because of the extra detection layer that contains extra three anchor boxes. In addition to that, YOLOv5-s6 outperforms YOLOv5-s in terms of mAP, recall, and precision with a mAP that is 1.5 percent higher, which shows that YOLOv5-s6 is more balanced than YOLOv5-s in term of precision and recall.

After the validation phase, we compared the models that were used previously on the test image dataset. In this experiment, we use the two models with and without TTA; the results are shown in Table 3. We observe in the validation phase that YOLOv5-s6 gives good results, and the last one gives good results either in the test phase or in the analysis phase, but to improve the results in this phase, we apply TTA to optimize the results.

**Figure 16:**
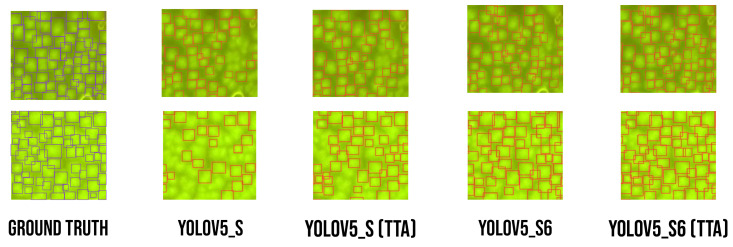
Comparing results of images from test phase with ground truth annotation.

In Figure 16, two examples of images are presented to illustrate the excellent detection results. The reason why using TTA impacts the result dramatically was because TTA applies various scale transformations with the following values [2,1,0.83,0.63] that help detect cells with different input sizes and select the best results with a voting algorithm, as shown in Figure 16. However, as we can see in Table 3, TTA has a direct impact on increasing the inference time.

#### 3.4.1. Models Comparison

In this section, we compared different models on the SARS-CoV-2 fluorescent RNA dataset using the mAP@0.5 metric. As shown in Table 4, we evaluated several popular detection models, including YOLOv5-s6, Faster R-CNN [38], Cascade R-CNN [39], Dynamic R-CNN [40], and PAA [41]. The results indicate that YOLOv5-s6 outperforms all other models, achieving a significantly higher mAP@0.5. This superior performance can be attributed to the use of anchor boxes, which are pre-defined bounding boxes of different shapes and scales to adapt to the size and shape of the SARS-CoV-2 fluorescent RNA in the image, in addition to applying Nonmaximum Suppression to filter out overlapping predictions. This improves the model’s ability to detect small objects that may be obscured or partially hidden by larger fluorescent RNA. In contrast, models such as Faster R-CNN, Cascade R-CNN, and Dynamic R-CNN, which rely on region proposal methods, and PAA that use a probabilistic anchor assignment algorithm, may not be as effective in crowded fluorescent cell images as they struggle to accurately identify and separate distinct SARS-CoV-2 fluorescent RNA when they overlap.

#### 3.4.2. Comparison of Different Backbones

In this section, we compared the YOLOv5 CSPnet backbone with different backbones, as Table 5 presents. We noticed that the YOLOv5-s6 with the CSPNet backbone gave the best results in terms of mAP@0.5. This phenomenon can be attributed to the presence of redundant gradient information during network optimization, leading to a significant reduction in complexity without compromising accuracy.

#### 3.4.3. YOLOv5 Variant Test

We applied a different variant (see Table 6) of YOLOv5 to compare the mAP with different backbone depths and with the presence of TTA. We find that depth plays a major role in getting a better mAP, with the trade-off of getting a large model that needs a great amount of computational power and with the consideration that we are going to use the model in a mobile application. We have chosen to use the YOLOv5-s6 because it has a good balance between accuracy and model size.

## 4. Conclusions

In this work, we proposed a simple but efficient approach, YOLOv5-s6, to detect SARS-CoV-2 fluorescent cells. The approach is based on a preprocessing step to extract the ROI and apply YOLOv5-s6 on this ROI to detect the fluorescent cells. The results show that our proposed approach works well with SARS-CoV-2 fluorescent cells. The paper addressed different variants and backbones of the YOLO detector. The TTA, combined with YOLOv5-s6, which has four detection layers (small, medium, large, and xlarge), has proved effective in detecting different scales of fluorescent cells. We should point out that our proposed approach to this investigation is still in its early stages, as illustrated by the limitations listed below:

1. We cannot compare our work with the state of the art because there is no recent work that tackles the detection of fluorescent cells from portable mgLAMP devices using a smartphone camera.
2. The model performed poorly in the case of a lot of crowded small cells in one test sample with more than 500 cells in one plastic frame.
3. Sometimes, the preprocessing method fails to extract ROI due to different lighting conditions and noise.

## 5. Future Work

In our future work, we will apply generative adversarial networks to solve the problem of small datasets, in addition to improving region of interest extraction and the preprocessing phase. We intend to use various pruning and quantizing techniques to deploy the model into a mobile application that will be accessible to low-end devices. 

## Figures and Tables

**Table 1:** Hyperparameters values.

Hyperparametres	Values
Input size	640×640×3
Batch size	32
Warmup_Epochs	3
Epochs	300
Scale	0.5
Mosiac	1
Mixup	0
Translate	0.1
$Anchor_{t}$	4
$HSV_{s}$	0.7
$HSV_{v}$	0.4
Momentum	0.937
Decay	0.0005

**Table 2:** YOLOv5 Performance on validation dataset.

Module	Input	mAP@0.5	Recall	Precision	Size
YOLOv5-s	640 × 640	94.1	91.2	91.5	12 Mb
YOLOv5-s6	640 × 640	**95.6**	**91.8**	**93.8**	25 Mb

**Table 3:** Performances results using YOLOv5-s and YOLOv5-s6 on test images.

Module	Input	mAP@0.5	Recall	Precision	Size	Inferance
YOLOv5-s	640 × 640	87	82.3	89	12 Mb	5.1 ms
YOLOv5-s (TTA)	640 × 640	88	83.5	90.2	12 Mb	7.9 ms
YOLOv5-s6	640 × 640	88.3	84.8	85.3	25 Mb	5.6 ms
YOLOv5-s6 (TTA)	640 × 640	**90.3**	**85.2**	**90**	25 Mb	7.0 ms

**Table 4:** Comparison of different models on the SARS-CoV-2 fluorescent RNA dataset.

Module	Backbones	mAP@0.5	Size
Dynamic R-CNN	Resnet50 [42]	50.3	330 mb
Faster R-CNN	Resnet50	58.7	320 mb
Cascade R-CNN	Resnet50	61.7	571 mb
PAA	Resnet50	70.4	256 mb
YOLOv5-s6	CSPnet	**90.3**	25 mb

**Table 5:** A comparison between different backbones.

Module	Backbone	Input	mAP@0.5	Size
YOLOv3-tiny	Darknet	640 × 640	79.1	33.2 Mb
YOLOv5-mobile	MobilenetV3 [43]	640 × 640	82.9	6 Mb
YOLOv5-s6	CSPnet	640 × 640	**90.3**	25 Mb
YOLOv5-VGG	VGG [33]	640 × 640	80.1	13.7 Mb
YOLOv5-Shuffle	Shuffelnet [44]	640 × 640	76.2	8 Mb

**Table 6:** Performance of YOLOv5-P6 variations.

Module	mAP@0.5	Size
YOLOv5-n6	83.3	6.29 mb
YOLOv5-s6	90.3	24 mb
YOLOv5-m6	91.2	71 mb
YOLOv5-l6	**91.6**	145 mb
YOLOv5-x6	88.9	267 mb

## Data Availability

Not applicable.

## Abbreviations

The following abbreviations are used in this manuscript:

AP	Average Precision
BN	Batch Normalization
CSP	Cross Stage Partial
CIoU	Complete Intersection over Union
DNA	Deoxyribonucleic acid
DIoU	Distance Intersection over Union
FN	False Negative
FP	False Positive
GFC	Green Fluorescent Cell
GIoU	Generalized Intersection over Union
GT	Ground Truth
IoU	Intersection over Union
LBP	Local Binary Patterns
LoG	Laplacian-of-Gaussian
mgLAMP	Membrane-Based In-Gel Loop-Mediated Isothermal Amplification
mAP	mean Average Precision
PCR	Polymerase Chain Reaction
PAnet	Path Aggregation Network
RNA	Ribonucleic acid
RCNN	Region-Based Convolutional Neural Network
SSD	Single-Shot Detector
SPP	spatial pyramid pooling
SPPF	Spatial Pixel Pair Feature
SGD	Stochastic gradient Descent
TTA	Test Time Augmentation
TP	True Positive
TN	True Negative
WBE	Wastewater Based Epidemiology
YOLO	You Only Look Once
